# Talking the Walk (Along): Lessons Learned From Engaging With Children With Cerebral Palsy and Their Parents for Investigating Lived Experiences of Falls

**DOI:** 10.1111/hex.70497

**Published:** 2025-11-14

**Authors:** Rebecca L. Walker, Thomas D. O'Brien, Gabor J. Barton, Bernie Carter, David M. Wright, Richard J. Foster

**Affiliations:** ^1^ Research Institute for Sport and Exercise Sciences Liverpool John Moores University Liverpool UK; ^2^ Faculty of Health, Social Care and Medicine Edge Hill University Ormskirk UK; ^3^ North West Movement Analysis Centre Alder Hey Children's NHS Foundation Trust Liverpool UK

**Keywords:** cerebral palsy, engagement, falls, walk‐along interview

## Abstract

**Background:**

Children with cerebral palsy (CP) regularly fall over, but causes of day‐to‐day falls are not well understood. Further insight may be revealed by engaging with children with CP and their families during patient and public involvement and engagement (PPIE) and adopting a participatory, child‐centred perspective. PPIE involves designing, conducting and disseminating research with the public and has been used in health research with children, but has not been utilised to inform research of falls with children with CP.

**Objective:**

This paper aims to critically discuss experiences of PPIE with children with CP and their parents, who engaged with a researcher to inform a novel adaptation of the walk‐along interview method for investigating how real‐world falls occur.

**Methods of Engagement:**

Eight children with CP (8–17 years) and six parents engaged as PPIE participants in consultations and activities with the researcher about a walk‐along interview method, specifically tailored to children with CP.

**Outcomes of Engagement:**

PPIE participants identified places to walk (e.g., parks), how to conduct interviews (e.g., ‘stop and talk’) and areas of questioning, that contributed to a walk‐along interview protocol. These outcomes demonstrate that PPIE generated unique insights for a protocol specific to children with CP.

**Discussion:**

Strength was brought to PPIE through developing good relationships and using creative activities. Challenges during PPIE included contrasting views and availability, which were managed through adaptation, communication and consensus.

**Conclusions:**

This study supports and expands previous PPIE and child‐centred work, reinforcing that children and parents can positively help create impactful research designs, by developing and refining a method to collect real‐world information about falls, specifically tailored for children with CP. We offer critical reflections on conducting PPIE, showing that engaging in PPIE to refine a protocol can offer unique insight into the worlds of children with CP and strengthen the design of future studies.

**Patient or Public Contribution:**

Children with CP and their parents were consulted using PPIE to provide their views about a novel walk‐along interview method tailored for children with CP. This paper focuses on lessons learned from this PPIE, which is part of a wider pre‐defined PhD project investigating causes of falls in children with CP. Within the wider project PPIE is an ongoing process beyond the scope of this paper.

## 1 Background

Cerebral palsy (CP) is a complex neuro‐musculo‐skeletal disorder caused by damage to the immature brain [1, 2, 3]. It is one of the most common causes of childhood motor disability and often leads to impaired balance and stability [1, 2]. Children with CP experience regular falls, 35% report falling daily, a further 30% report falling weekly [4]. These falls occur throughout childhood for children with CP, up to 18 years of age [4], which can have significant psychosocial effects on childhood wellbeing, for example, reduced balance confidence, reduced activity participation, increased feelings of fear, embarrassment and frustration [5, 6]. Typical assessment of children with CP is done over level ground [2], and therefore may not provide an accurate representation of stability, and therefore fall risk in outdoor, real‐world environments [7]. Previous investigations have demonstrated that instabilities exist for children with CP during activities designed to replicate challenging day‐to‐day places [8, 9, 10]. However, there are still limited links between challenging environments and real‐world falls [11]. The lack of knowledge of falls and fall risk in children with CP could be because the environments that children tell us are likely to cause falls, based on their everyday lived experiences, have not yet been investigated. Identifying where and how falls happen through scientific study relies on known truths about how children with CP experience the world around them. For example, assuming that a step up would cause instability and thus high fall risk, when perhaps children would ask for help or avoid walking up steps in the real‐world.

Patient and public involvement and engagement (PPIE) is commonly used to gain input on research from outside the research team on how research is designed, conducted or disseminated [12, 13, 14]. PPIE has been successful with children with chronic conditions and disabilities, including CP [15, 16, 17, 18, 19], and this has positively informed research about their day‐to‐day lived experiences. Furthermore, Lansdown [20] expresses the significance of hearing children's voices in research, taking on board children's views as important and unique, and regarding them as experts of their own experiences. PPIE provides the opportunity for children with CP and their parents to feel valued in research that concerns them, in a more novel way than they may be used to, for example, being involved clinical scientific studies. Key literature emphasises that children and young people should have a say in research [13, 21, 22], including specifically those with disabilities [16, 17, 18, 19]. PPIE has the potential to explore authentic lived experiences of children with CP and their parents or guardians to increase applicability and practicality of a research protocol for a novel purpose and specific research group (falls in children with CP). By using child‐centred methods, further insight into the causes of falls in children with CP in real‐world environments can be revealed. This knowledge is fundamental to design future work that could help us learn more about falls and how to prevent them.

PPIE in this paper involved consulting with children with CP and their parents to inform decisions about a scientific protocol for understanding lived experiences of falls. Specifically, the PPIE reported in this paper focused on refining a walk and talk interview technique [23], for use within a study investigating the causes of day‐to‐day falls in children with CP. Walk and talk interviews are a method by which a participant and researcher take a walk and engage in a conversation based on environmental cues around them [23]. This method has shown to offer rich insights into how people experience the world around them [24, 25, 26, 27]. Although the researchers brought key ideas (e.g., the concept of walk and talk interviews being conducted in the real‐world) to the PPIE discussions, the children and their parents were key to informing and shaping the methods. A safe and supportive space was created by using a transparent set of PPIE values specific to our work, that informed all interactions (Table 1).

**Table 1:** PPIE values specific to this study and examples of how these were instilled during PPIE.

Value	Meaning (specific to this project)	Example within PPIE	
		Child	Parent
Child‐centred	Keeping the child's thoughts and experiences at the heart of decision making	Creative tasks and one‐to‐one conversations were used for informing the protocol, which allowed children to have their individual voices heard without prompt or interruption	Parents were consulted about the child‐centred aims of the project and were provided the opportunity to expand and discuss the thoughts of children
Safe space	Having a comfortable place for open conversation about daily life experiences and falls	PPIE was hosted at Stick ‘n’ Step, a local charity that children regularly attended and therefore already have regular conversations about walking, cerebral palsy and falls in this environment	Parents asked for, and were provided with, the option to chat over the phone about their child's previous experiences, offering both convenience for time and the ability to talk in their own home
Right to engage	Ensuring that children and parents know they can be involved with PPIE as little or as much as they wish	Volunteering took place at the local charity for months before PPIE, to allow children to be comfortable with the research and in choosing whether they would like to participate	Parents were familiar with the researcher before PPIE participation due to volunteering. Flyers were requested by parents before participating, which were then provided to give full information.
Flexibility and responsiveness	Allowing children and parents freedom to shape the project with their opinions	Parents and children had freedom to shape the project. For example, if child and/or parent engaging with PPIE did not like our pre‐determined ideas, we would discuss and consider changes.	
Respect	Acknowledging the voices and opinions of children and parents	Follow up discussions were had with both children and parents after making decisions about the protocol to ask if the decisions were representative of PPIE consultations. Additionally, occasional contradictory opinions of children and parents were addressed directly.	

This paper aims to evaluate and critically reflect on the process and outcomes of using PPIE with children with CP and parents of children with CP who engaged with a researcher to share ideas. In particular, this paper shares how insights from both children and parents were crucial in refining the development of a walk‐along interview protocol, specifically tailored for children with CP.

## 2 Methods of Engaging With Children and Parents

This paper is presented in accordance with the GRIPP‐2 [28] (Supporting Information: S1).

### 2.1 How Did We Recruit Children and Parents to Engage in Our Ppie?

The lead investigator (RW) volunteered at Stick ‘n’ Step [29], a charity for children with CP and their families local to the Liverpool City Region, for 125 h across 8 months. Parents of children who attend Stick ‘n’ Step, and their children were told about the project and how they could help if they wished to take part in PPIE.

Ethics approval is not required for PPIE consultations; however, this study abided by typical PPIE ethical considerations and principles [30]. This included (1) building transparent relationships (children and parents being aware of the PPIE and research), (2) volunteering kept separate from PPIE activity, (3) presence of a familiar member of staff at Stick ‘n’ Step, (4) fully informing children and parents of the purposes of PPIE in advance of any PPIE consultations and (5) having anonymity and confidentiality in consultation outcomes. Moreover, children and parents were reassured they could engage as much or as little as they wished and were under no obligation to engage and could stop engaging at any point. The lead investigator had appropriate safeguarding and training as a requirement for volunteering for example, DBS check. Permission was granted verbally from parents for children to engage in PPIE consultations.

### 2.2 Who Did We Engage With, When and Where?

Eight children with CP (aged 8–17 years old) and six parents of children with CP (not all related to the participating children) decided to engage over 6 weeks. The lead investigator spoke to six children on one occasion and to two children on two occasions (total of 10 1‐to‐1 chats). Three of the eight children also participated in a group session asking about the small portable cameras that were planned for use in the walk and talk interviews. The children tried this equipment and provided feedback. Six parents engaged in consultations: two parents opted for face‐to‐face and four opted to talk by telephone.

**Figure 1:**
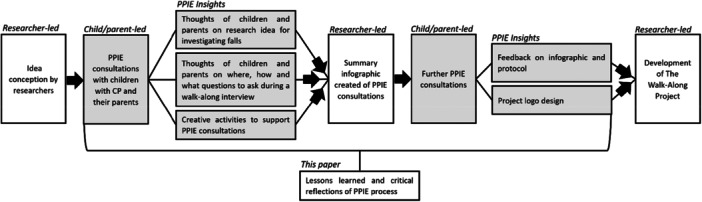
Schematic of PPIE process. Grey boxes indicate child and parent involvement.

Following initial consultations, the research team's draft ideas for the ‘walk and talk’ method were taken back to several children and parents for further consultation. This was presented as an infographic that addressed every element that the research team had learned, based on notes taken and activities conducted during PPIE. Children and parents were shown the infographic and given the opportunity for further feedback and engagement and to ensure we had effectively captured their thoughts and opinions within our protocol. In this second round of consultation, children additionally contributed to the design of a logo for the study. A schematic of the PPIE process and how children and parents were involved in the development of the walk and talk method of data collection for investigating falls is represented in Figure 1.

### 2.3 How Did We Engage With Children and Parents?

Consultations with children and parents lasted approximately 15 min; consultations with the children were supplemented with creative activities, described below. Consultations started by talking about falls, with the children and parents giving a general opinion on whether falls were an issue in day‐to‐day life followed by questions about whether they thought walk and talk interviews were a good idea and whether they thought it would be a practical approach to use.

Consultations were guided by three main PPIE objectives to learn from children with CP and their parents: (i) *where* interviews should take place (based on conversations about children's daily walking places and previous experiences of falls), (ii) *how* to conduct safe and insightful interviews outdoors with children with CP (based on conversations of what would make children and parents feel comfortable while also getting an accurate representation of day‐to‐day life) and (iii) *what questions* they think need to be asked during interviews to understand how falls occur in the real‐world (based on conversations about day‐to‐day, real‐world experiences of falls). Activities were designed to enhance PPIE consultations.

### 2.4 Activities

#### 2.4.1 Photo Activity: Describing Challenging Places

**Figure 2:**
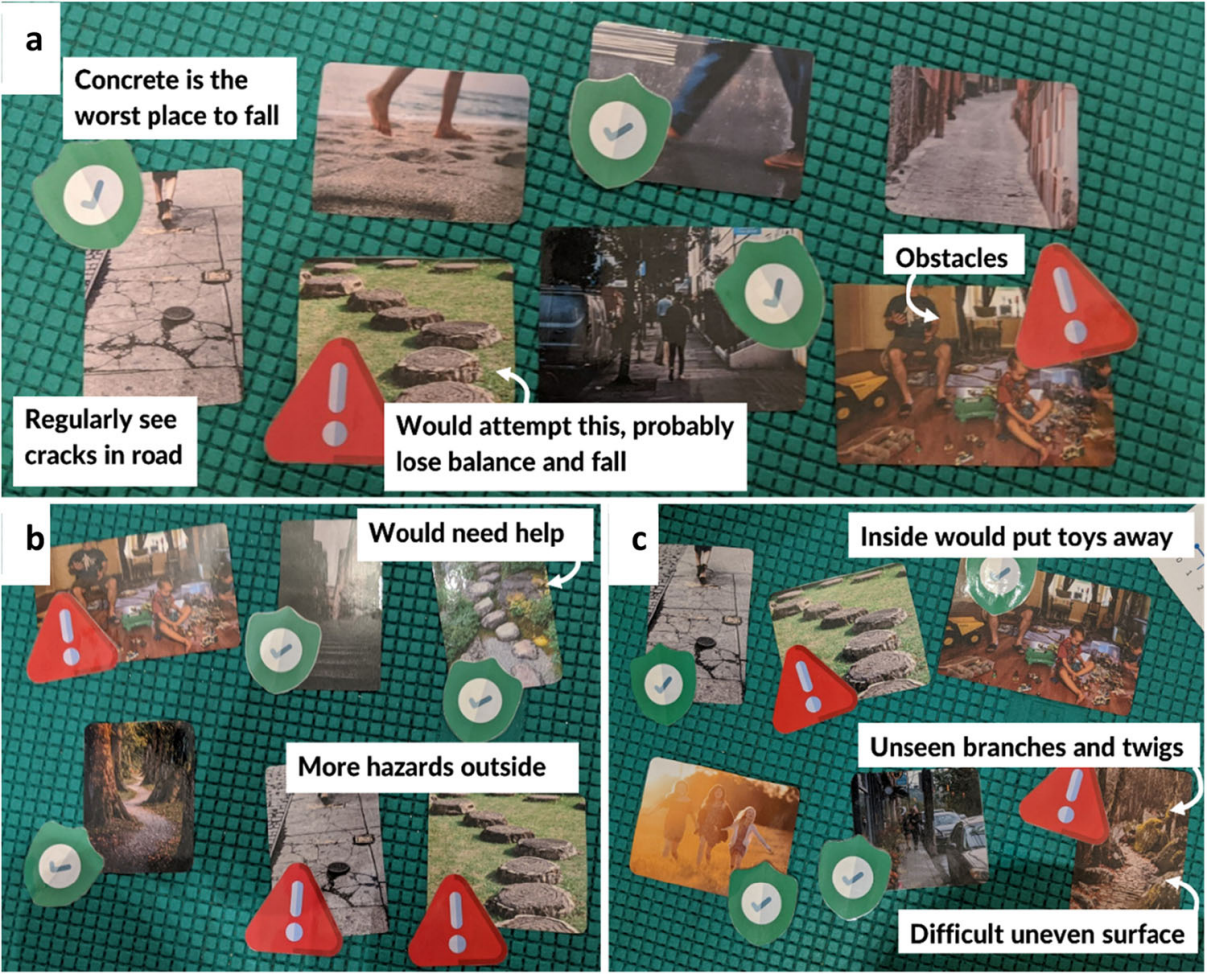
Three examples (a–c) of an activity used during separate consultations with three children. Annotations are researcher notes interpreted from conversations had during PPIE consultations about each photo. Photographs sourced from Microsoft Stock Images, Stickers sourced from Flaticon.com.

The first child‐centred activity used images (Microsoft Word Stock Images, Microsoft 365, Microsoft Corporation Washington, USA) chosen by the research team to reflect everyday environments that the researchers believed may pose a fall risk. Images were selected based on previous work that suggests children with CP show stability differences compared to TD children over uneven surfaces [8]. Images were used that showed both level (e.g., a concrete path) and uneven environments (e.g., a cobbled path) that children might face in the real‐world (e.g., on the way to school). The lead investigator presented a random selection of six to eight images to the children and asked them to place stickers onto the photographs, using red stickers for places they believed could cause a fall or green stickers for somewhere safe. Children were then asked why they had placed red or green stickers each photograph; this led to detailed conversations around what may cause a trip or a fall in the real‐world and that this should be considered in our walk and talk interview design. An example of how these detailed conversations occurred during this first activity can be seen in Box 1. At the end of each conversation, a photograph was taken of all the stickered images and this was then annotated to summarise the conversation. Figure 2 shows an example of this activity method.

Box 1 **Example unique insight from creative methods.**

Children were presented with several photographs. One (see below) showed a child indoors with many toys around the room.

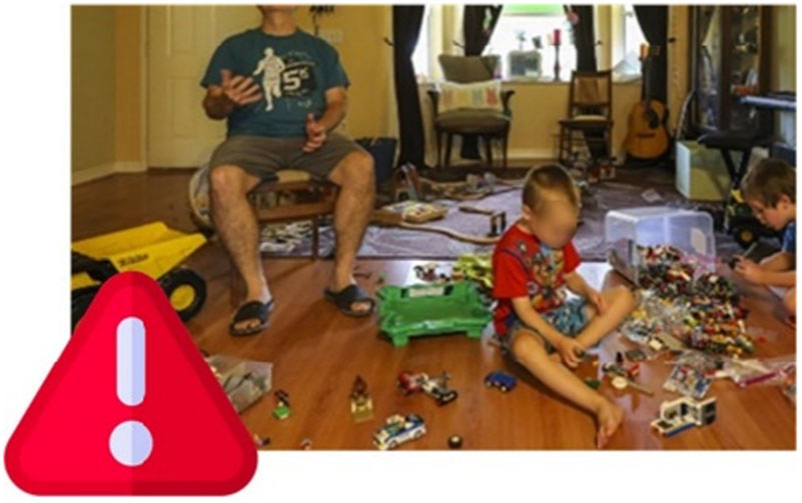

Children were consulted about why they had chosen to place a red sticker on this photo. For example, the red sticker indicated that this is somewhere with high fall risk, however in further conversation, children explained that this would not typically cause a fall in each children's own home, because they would tidy their own toys away to avoid the risk.

#### 2.4.2 Logo Activity: Bringing the Project Together, Together

**Figure 3:**
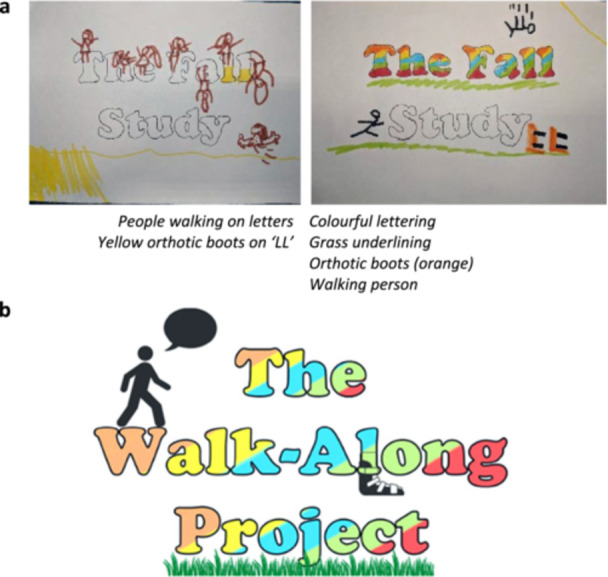
(a) Children's initial drawings with key elements written underneath that were taken to inform the final logo and (b) final logo that contained person walking, grass underlining, orthotic boots on the letter ‘L’ and with colours that appealed to one of the children.

The final element of the PPIE activity involved five children helping to develop a logo. Children were given a list of potential study names and invited to colour in and add some drawings to the names. The children drew stick figures walking and standing and falling over, orthotic boots, splints and grass (Figure 3a). Some children chose not to help with the design, deciding to instead draw a train or colour in letters. One child was shown the logo and asked to describe what they would include, rather than write on the logo by their own choice and they suggested that the logo should include images of people, grass and a football. The children chose the name of the study—The Walk‐Along Project. Following the ideas presented by children, based on the rainbow colours they liked and used, and the core images they wanted, the lead investigator wove these ideas into the final logo design (figure 3b). The logo represents the involvement of the children's lived experiences and ideas.

## 3 Outcomes of Engagement

The primary outcome of this engagement work was that PPIE consultations helped inform several aspects of a walk‐along interview protocol to be used for investigating children's lived experiences of falls in the real‐world. Impacts, strengths and weaknesses of PPIE within the current study are considered in detail in the discussion.

**Figure 4:**
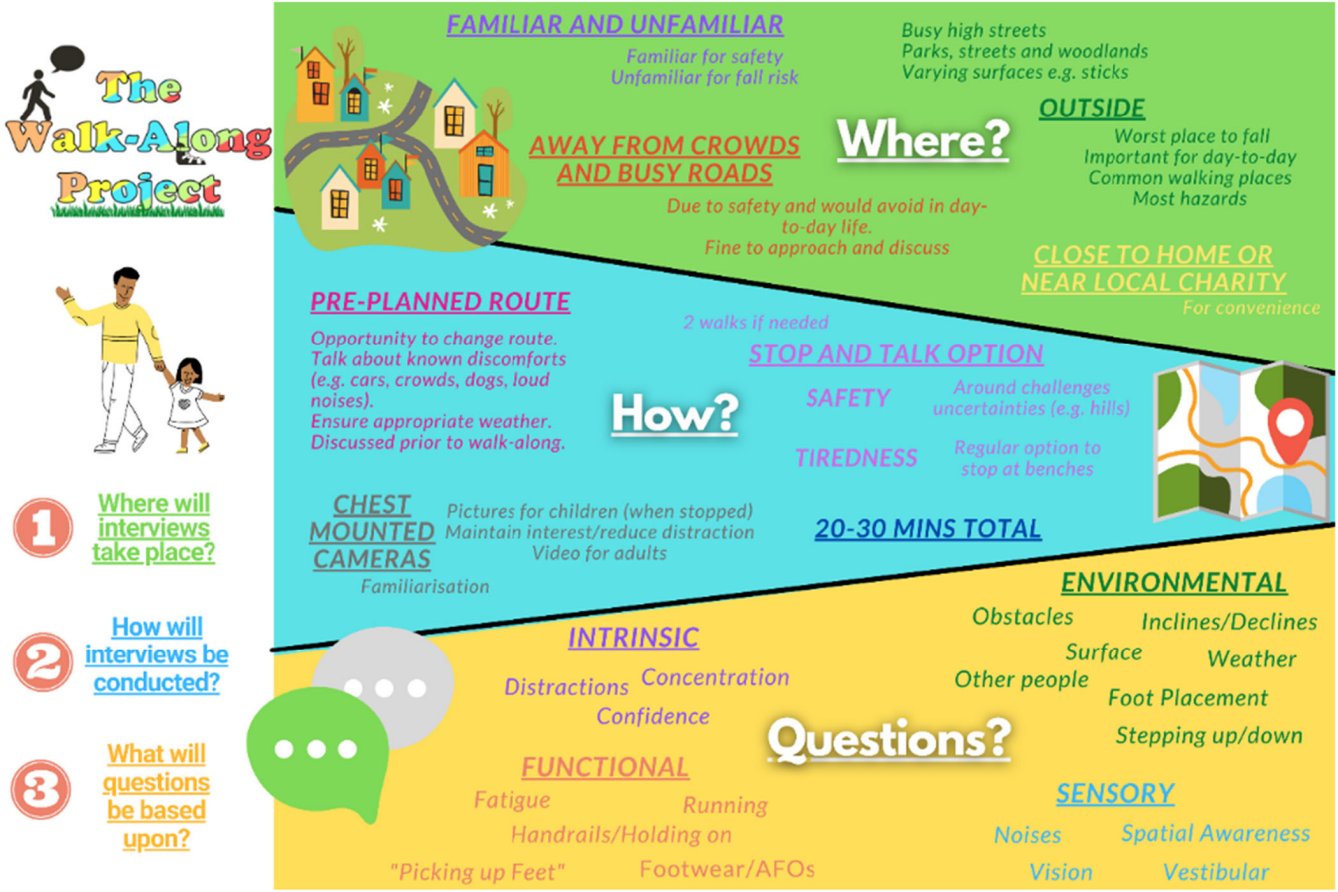
Infographic representing a summary of all information shared during PPIE consultations, to inform (1) Where interviews should take place, (2) How to conduct safe and insightful interviews and (3) Areas of questioning during interviews.

Children and parents responded positively when asked about using walk and talk interviews to look at real‐world fall risk. They suggested it would be easier to recall how falls happen in the real‐world day‐to‐day if they were talking about them in those environments that might typically cause a fall. Children and parents suggested that walk‐along interviews should be conducted somewhere convenient (e.g., close to home) and where they typically walk (e.g., to school or the park). This was supplemented by the photo activity described earlier. Children and parents additionally suggested how to conduct interviews (e.g., having regular breaks to stop and talk about surroundings and taking photos of places that are most challenging to maintain engagement). Finally, children and parents talked about what they thought caused day‐to‐day falls, which helped us to design questions and be aware of factors relevant to the environments in which walk and talk interviews would take place. An infographic was developed to summarise all information shared during our PPIE consultations (Figure 4).

The term ‘walk and talk’ interviews which we used in the PPIE is just one of a range of different terms (e.g., walking interviews [27, 31], go‐along interviews [23, 32], walking‐whilst‐talking method [33] or walk‐along interview [34]) used in the literature to describe the same method. After the suggestion from children and parents that they would prefer to stop and talk during the walk, to be able to observe surroundings, rather than continually walking and talking during the interview, we decided to adopt the term walk‐along interviews.

A measure of the success of PPIE in developing the protocol and the walk‐along interviews is evident in the recruitment of 12 children and their parents to The Walk‐Along Project. Success is also reflected in the positive feedback and enjoyment from the children and parents who participated in the PPIE and The Walk‐Along Project. The Walk‐Along Project has generated rich child‐centred data sets that reflect deep insights into children's understandings of everyday falls and which has an extensive rich resource of photographs and videos of the places where falls occur and the challenging walking environments faced daily by children with CP.

## 4 Discussion

The aim of our PPIE was to produce a child‐centred, engaging method for data collection that would encourage children to participate. Working with children and parents we developed a walk‐along interview method specifically for children with CP and their parents and the real‐world challenging environments they face to help us investigate the causes of day‐to‐day falls in children with CP. We wanted to learn about the thoughts and experiences of children with CP and their parents. In the following discussion we evaluate and critically reflect on the process and outcomes of PPIE with children with CP and their parents.

### 4.1 Impact of PPIE

Historically, falls and stability research addressing children with CP, uses quantitative assessments of gait [8]. To the best of our knowledge, we are the first to use and share robust PPIE work that draws on the thoughts and experiences of children with CP to shape a novel walk‐along interview protocol for investigating everyday causes of falls. Specifically, our PPIE work has used insights from children with CP and their parents to improve (1) our protocol's applicability (e.g., enabling us to discuss experiences in known high‐risk places) and (2) feasibility (e.g., stopping to talk rather than walking while talking). Definitions provided earlier for PPIE were appropriate and consistent for this specific PPIE. PPIE narrowed the focus of the walk‐along interview protocol to the most important considerations for causes of falls in the real‐world. The walk‐along interview method we developed extends walk‐along methods that have been used with older adults [24, 25, 26] and TD children [32, 34, 35]. Sharing the lessons learned from PPIE in this study hopefully offers further evidence for the use of PPIE in health research and with children [13, 18, 36].

### 4.2 Strengths and Challenges of PPIE

Our PPIE was successful due to three core child‐centred factors. First, relationships and trust with children and parents were built by the lead investigator volunteering before the start of PPIE. This helped children to be comfortable in talking and allowed the lead investigator to develop deeper insight and understanding of the lives of children with CP. Second, creative methods used during PPIE consultations offered children interesting, child‐centred and informal opportunities to provide unique insights specific to their lives and interests. This strength is consistent with past engagement work using creative methods [37, 38]. Third, PPIE was clearly informed and underpinned by a strong value base that was important to recognise and respect children's capacity and contributions (see Table 1) [20]. Adopting a flexible consultation approach (e.g., phone calls or in person) was additionally crucial to allow involvement of children and young people with disabilities, aligning with previous work [18].

Challenges to PPIE included distractions during consultations and availability. These were overcome through use of creative activities and flexible scheduling of consultations. A final challenge faced was contrasting opinions. For example, when one child explained that they ‘never’ fall over, their parent then reminded them that they had fallen getting out of the car that morning. This example demonstrates the importance of gaining context regarding why these contrasting opinions occurred, allowing us to ensure that the views of children and parents were equally integrated into our research design.

### 4.3 Lessons Learned and Limitations

Finding the balance of what are ‘safe’ and what are perceived as high fall risk places was learned during consultations and protocol design. Ensuring interviews would be ‘safe’ may have constrained the use of riskier locations. However, if children and parents were unsure about safety, we were either unlikely to have generated the rich insights we gained during the research interviews that followed this PPIE, or they may have declined to participate. In conducting PPIE, we discussed with parents and children additional measures we could use that would allow interviews to be conducted safely, without missing potentially important information about day‐to‐day situations that might increase a fall.

It is also important to acknowledge that the decision to avoid places deemed to be unsafe was done so following consultations with parents who explained that although they would typically avoid such places, they would be happy to discuss these places during the walk‐along interviews.

A lesson learned from using creative activities were that photographs used during the photo‐sticker activity (Figure 2) did not necessarily align to places children encountered in their own day‐to‐day lives (e.g., Box 1 example), so although places were identified as high fall risk, it may not be something they themselves struggle with day‐to‐day. Using photos as a prompt for conversation rather than at face‐value was an important lesson as we were able to explore not just where children thought their fall risk might increase, but why they thought this.

A key limitation of this PPIE is the lack of ethnic and gender diversity across PPIE participants. A more diverse group of children and parents could have shared different perspectives on daily life, this is noted elsewhere as something that should be acknowledged for future work [18]. Secondly, our PPIE participants were all approached through the same charity that among other things involves stability tasks and fall avoidance [39]. However, accessibility to this charity is not readily available for all children with CP, so our PPIE is not representative of the wider population of children with CP.

Perhaps another limitation to our PPIE is that although our engagement with children and parents was respectful, collaborative and committed, our engagement is best described as researcher‐ rather than child‐led as we brought some established ideas to our PPIE. The role of PPIE participants was to help develop and refine a child‐centred, engaging, participatory method (the walk‐along interview); they started with an outline of the method created by the research team, including ideas for the study name. Despite these limitations and our acknowledgement of our PPIE being researcher‐led, there was a considerable amount of engagement and children's perspectives informed the study protocol.

## 5 Final Thoughts and Conclusions

Meaningful PPIE with children and young people can be considered an important part of research. This paper tells a story of how child‐centred PPIE with children with CP and their parents meaningfully contributed to a research design and discusses the strengths and challenges in doing so. Our PPIE was greatly strengthened due to the amount of time dedicated to forming strong, trusted relationships in the community through volunteering. Overall, our PPIE with children with CP and parents of children with CP, helped strengthen, and refine a method to collect real‐world information about falls, specifically for children with CP, in an area with limited reported use of PPIE or consideration of children's views in research and methods design. A measure of the value of our PPIE process is we have now successfully used the walk‐along interview method with children with CP to reveal deep novel insights into the causes of falls in the real‐world.

## Author Contributions

**Rebecca L. Walker:** validation, formal analysis, investigation, resources, data curation, methodology, writing – original draft, writing – review and editing, visualization, project administration. **Thomas D. O'Brien:** conceptualization, validation, methodology, writing – review and editing, supervision. **Gabor J. Barton:** conceptualization, validation, methodology, writing – review and editing, supervision. **Bernie Carter:** conceptualization, validation, data curation, methodology, formal analysis, visualization, writing – review and editing, supervision. **David M. Wright:** conceptualization, validation, writing – review and editing, supervision. **Richard J. Foster:** conceptualization, methodology, formal analysis, data curation, writing – review and editing, visualization, supervision, project administration, funding acquisition.

## Ethics Statement

In line with requirements of the Health Research Authority, ethics approval is not required to conduct PPIE. This paper reports PPIE, not research, and no information collected allocated can be linked to specific PPIE participants, thus no ethics approval was required. Permission was granted verbally from parents/guardians for children to engage in PPIE conversations. The information provided in this paper has been guided by the Guidance for Reporting Involvement of Patients and the Public (GRIPP) 2, long form (Staniszewska et al. [28]) (Supporting Information: S1).

## Conflicts of Interest

The authors declare no conflicts of interest.

## Supporting information


**Supplementary Material 1:** Guidance for Reporting Involvement of Patients and the Public (GRIPP) 2 checklist, long form2.

## Data Availability

Data sharing not applicable to this article as no data sets were generated or analysed during the current study.
